# Endocrinological and Cardiological Late Effects Among Survivors of Childhood Acute Lymphoblastic Leukemia

**DOI:** 10.4274/Tjh.2012.0094

**Published:** 2013-09-05

**Authors:** Pakize Karakaya, Şebnem Yılmaz, Özlem Tüfekçi, Mustafa Kır, Ece Böber, Gülersu İrken, Hale Ören

**Affiliations:** 1 Department of Pediatrics, Dokuz Eylül University Faculty of Medicine, İzmir, Turkey; 2 Department of Pediatric Hematology, Dokuz Eylül University Faculty of Medicine, İzmir, Turkey; 3 Department of Pediatric Cardiology, Dokuz Eylül University Faculty of Medicine, İzmir, Turkey; 4 Department of Pediatric Endocrinology, Dokuz Eylül University Faculty of Medicine, İzmir, Turkey

**Keywords:** Cardiotoxicity, Chemotherapy, childhood, Endocrinology, Leukemia, Late effects

## Abstract

**Objective:** Survival rates for childhood acute lymphoblastic leukemia (ALL) have significantly improved and late effects of therapy have been important in the follow-up of survivors. The objective of this study is to identify the endocrinological and cardiological late effects of ALL patients treated in our pediatric hematology unit.

**Materials and Methods:** Patients treated for ALL with BFM protocols after at least 5 years of diagnosis and not relapsed were included in the study. Endocrinological late effects (growth failure, obesity, insulin resistance, dyslipidemia, thyroid gland disorders, osteopenia/osteoporosis, and pubertal disorders) and cardiological late effects were evaluated. The study group was evaluated with anthropometric measurements, body mass index, and laboratory testing of fasting glucose, insulin, serum lipids, thyroid functions, and bone mineral densities. Echocardiography and pulsed wave Doppler imaging were performed for analysis of cardiac functions.

**Results:** Of the 38 ALL survivors, at least 1 adverse event occurred in 23 (60%), with 8 of them (21%) having multiple problems. Six (16%) of the survivors were obese and 8 (21%) of them were overweight. Subjects who were overweight or obese at the time of diagnosis were more likely to be overweight or obese at last follow-up. Obesity was more frequently determined in patients who were younger than 6 years of age at the time of diagnosis. Insulin resistance was observed in 8 (21%) subjects. Insulin resistance was more frequently seen in subjects who had family history of type 2 diabetes mellitus. Hyperlipidemia was detected in 8 (21%) patients. Hypothyroidism or premature thelarche were detected in 2 children. Two survivors had osteopenia. Cardiovascular abnormalities occurred in one of the subjects with hypertension and cardiac diastolic dysfunction.

**Conclusion: ** We point out the necessity of follow-up of these patients for endocrinological and cardiological late effects, since at least one adverse event occurred in most of our cases.

**Conflict of interest:**None declared.

## Abstract (tr)

**Amaç:** Akut lenfoblastik lösemili (ALL) çocuklarda sağkalım oranı belirgin düzelmiş ve izlemlerde geç yan etkiler açısından değerlendirilmeleri önemli hale gelmiştir. Bu çalışmanın amacı çocuk hematoloji kliniğimizde tedavisi tamamlanan akut lösemi olgularında uzun dönemde gelişebilecek endokrinolojik ve kardiyolojik geç etkilerin belirlenmesidir.

**Gereç ve Yöntemler:** Lösemi tanısı aldığı tarihten itibaren en az beş yıl geçmiş, relaps gelişmemiş ve BFM protokolleri ile tedavi edilmiş hastalar endokrinolojik geç etkiler (boy kısalığı, obezite, insülin direnci, dislipidemi, tiroid hastalığı, osteopeni/osteoporoz, puberte sorunları) ve kardiyolojik geç etkiler açısından değerlendirildi. Çalışma grubunda antropometrik ölçümler, vücut kitle indeksi, açlık kan şekeri, insülin, serum lipid ve tiroid fonksiyon testleri ve kemik mineral dansiteleri araştırıldı. Kardiyak fonksiyonların analizinde ekokardiyografi ve pulsed Doppler görüntüleme kullanıldı.

**Bulgular:** 38 olgudan 23’ünde (%60) en az bir, 8 olguda (%21) ise birden fazla geç etki olduğu görüldü. Altı olgunun (%16) fazla kilolu, 8’inin (%21) obez olduğu belirlendi. Tanı anında fazla kilolu ya da obez olanlarda ve tedavi bitiminde fazla kilolu ya da obez olanlarda son değerlendirmede obezite daha yüksek orandaydı. Tanı anında altı yaşın altında olanlarda obezite daha fazlaydı. Sekiz olguda (%21) insülin direnci vardı. Obez ya da fazla kilolu olanlarda ve ailede tip 2 DM öyküsü bulunanlarda insülin direnci daha yüksek oranda bulundu. Sekiz olguda (%21) hiperlipidemi saptandı. Hipotiroidi veya prematür telarş birer olguda, osteopeni 2 olguda görüldü. Kardiyolojik geç etki olarak bir olguda hipertansiyon ve hafif diastolik disfonksiyon olduğu belirlendi.

**Sonuç:** Olguların çoğunda en az bir geç yan etki gözlendiğinden, bu çocukların endokrinolojik ve kardiyolojik geç etkiler açısından takip edilmelerinin gerekli olduğu ortaya çıkmaktadır .

## INTRODUCTION

Survival rates of children treated for acute lymphoblastic leukemia (ALL) have improved significantly over the last 4 decades. More than 85% of children with ALL are expected to become long-term survivors [1,2]. Many long-term survivors of childhood cancer will develop chronic physical or psychosocial problems as a result of their cancer or its therapy [3,4,5]. The follow-up of late effects has assumed increasing importance as there are large numbers of childhood leukemia survivors [6]. In this study we aimed to determine the endocrinological and cardiological late effects in childhood ALL survivors treated with BFM protocols in our pediatric hematology unit. 

## MATERIALS AND METHODS

**Patients**

The patients included in this single-center cross-sectional study fulfilled the following criteria: diagnosis of ALL in our center between January 1993 and December 2007; age under 18 years at the time of diagnosis; treatment in our center according to the ALL BFM-90 and ALL BFM-95 treatment protocols; no relapse, not including treatment with bone marrow transplantation; and a follow-up time of ≥5 years after the first diagnosis. In order to collect data the survivors over 18 years of age or the legal guardians of younger survivors were called by phone and informed about the study. Those who agreed to participate in the study were called to the outpatient clinic and informed consent was obtained. A total of 38 patients were included in the study. Data including information about age, sex, age at diagnosis, anthropometric measures at diagnosis, initial echocardiographic measurements, leukemia type, subtype and risk group according to BFM protocols, administered treatment protocols, history of central nervous system disease and cranial irradiation, details about date and dosage of cranial irradiation, cumulative anthracycline and steroid doses, and the time after diagnosis were obtained from the medical records of the patients. 

**ALL BFM-90 and BFM-95 Protocols**

The ALL BFM-90 treatment protocol was administered between 1993 and 1995 and the ALL BFM-95 was administered thereafter. In both the ALL BFM-90 and BFM-95 protocols, patients were assigned to a standard-risk group (SRG), medium-risk group (MRG), or high-risk group (HRG). Both protocols consist of 4 phases: induction, consolidation, reinduction, and continuation. In ALL BFM-90 and ALL BFM-95, the cumulative anthracycline doses were 180 mg/m2, 240 mg/m2, and 300 mg/m2 in the SRG, MRG, and HRG, respectively. In ALL BFM-90, preventive cranial radiotherapy was indicated for T-cell ALL and the MRG and HRG with a dose of 12 Gy. Therapeutic irradiation dose for patients with initial involvement of the central nervous system was 12 Gy for patients 1 to 2 years old and 24 Gy for patients of more than 2 years of age. In ALL BFM-95, preventive radiotherapy was only indicated for T-cell ALL and the HRG, and the central nervous system-directed therapy dose was reduced to 18 Gy from 24 Gy. Infants younger than 1 year were neither preventively nor therapeutically irradiated. The duration of treatment was 24 months for all patients in ALL BFM-90, 36 months for male MRG patients, and 24 months for all patients in “ALL BFM 95” [7]. 

**Evaluation of Endocrinological Late Effects**

The standing height of the survivors at the time of diagnosis and at the time of the study was noted. Height percentiles were defined using the Centers for Disease Control and Prevention (CDC) growth charts [8]. Height below the third percentile was accepted as short stature. Body mass index (BMI) of the patients at the time of diagnosis and at the time of the study was calculated as weight in kilograms divided by height in meters squared. BMI percentile for age and sex was determined using reference standards from the CDC [8]. Subjects with BMI at or above the 95th percentile for their age were defined as obese and those with BMI at or above the 85th percentile for their age were defined as overweight. For adult subjects, overweight was defined as a BMI of 25-29, whereas obesity was defined as a BMI of ≥30. Fasting insulin, glucose, and lipid levels were measured. The estimate of insulin resistance was calculated by the homeostasis model assessment (HOMA) index. A cut-off HOMA level of >2.5 in children and >4.0 in adolescents was used to identify insulin-resistance status [9]. Total cholesterol of >200 mg/dL and low-density lipoprotein cholesterol of >130 mg/dL were defined as high [10].

Cumulative steroid dose, history of cranial irradiation, familial history of type 2 diabetes mellitus, hyperlipidemia, and insulin resistance were all noted and tested for relationships with obesity. 

Thyroid function tests (free T4 and thyroid-stimulating hormone) were used for evaluation of thyroid gland disorders. Thyroid ultrasonography was performed when goiter was detected in physical examination or in the case of abnormal thyroid functions. 

Bone mineral density (BMD) was measured from the lumbar spine by dual energy X-ray absorptiometry. Age- and sex-based z-scores of BMDs indicating osteopenia or osteoporosis risks were calculated. Z-score values above -1 standard deviation (SD) were considered as normal, between -1 SD and -2 SDs as osteopenia, and below -2 SDs with clinically significant fracture history as osteoporosis [11]. 

Pubertal stages of the patients were assessed according to the Tanner stages [12].

Bone ages were evaluated according to the atlas of Greulich and Pyle [13]. Precocious puberty was defined as development of pubertal changes before 8 years of age in girls and 9 years of age in boys [14]. Delayed puberty was defined as the lack of any secondary sexual characteristics by the age of 13 years in girls and 14 years in boys [15]. If precocious puberty or pubertal delay was detected, further evaluations with measurement of follicle-stimulating hormone, luteinizing hormone, estradiol, and testosterone levels were performed. Menarche ages of female survivors were noted. 

**Evaluation of Cardiological Late Effects**

Systolic and diastolic blood pressure measurements were evaluated according to age, sex, and height. Blood pressure readings were classified as normal, prehypertensive, or hypertensive [16].

Cardiomegaly was defined as measurement of the cardiothoracic ratio at >50% on telecardiography. A standard 12-lead electrocardiography (ECG) was recorded and analyzed for rhythm disturbances and ventricular hypertrophy findings. The echocardiographic imaging was performed with a Philips IE 33 equipped with 1- and 5-MHz transducers. A standardized M-mode, 2-dimensional, Doppler echocardiogram examination was performed with multiple orthogonal parasternal, apical, and subcostal views with the patient in the left lateral decubitus position. All echocardiogram examinations and blood pressure measurements were performed by a pediatric cardiologist when patients were in resting position. To assess the capacity of the left ventricle, the following parameters were measured: left ventricular end-diastolic and end-systolic dimension and the septal and posterior wall thickness in diastole. Systolic function of the left ventricle was defined by 2 parameters: the ejection fraction and the fractional shortening. Diastolic function of the left ventricle was assessed with Doppler echocardiogram. Parameters included peak early mitral blood flow velocity (E) and peak mitral velocity during atrial contraction (A); the value for the E/A ratio was calculated. An E/A ratio of <1.00 was considered as abnormal with moderate diastolic dysfunction. Deceleration time of peak early blood flow velocity and isovolumic relaxation time were measured [17,18,19]. An ejection fraction of 60% or more was considered as normal [20].

**Statistics**

All statistical analyses were performed using SPSS 15. Categorical data were expressed as frequencies and percents and continuous variables were expressed as mean ± SD. Differences between groups for categorical variables were compared by chi-square and Fisher exact tests. For continuous variables, the Mann-Whitney U test was used. Values of p<0.05 were selected as reflecting statistical significance. 

## RESULTS

**Characteristics**

Of the 38 ALL survivors, 20 (53%) were females and 18 (47%) were males. The median age was 12 years (range: 7-28 years). They had been off therapy for an average of 8 years (range: 5-17 years, SD: 3.4 years). Table 1 shows the characteristics of the 38 patients. 

At least 1 adverse event occurred in 23 (60%) of the 38 survivors, with 8 of them (21%) having multiple problems (Table 2). 

**Endocrinological Late Effects**

Six (16%) of the survivors were obese and 8 (21%) of them were overweight. Subjects who were overweight or obese at the time of diagnosis were more likely to be overweight or obese at the last follow-up. Twenty-six percent of the patients with normal BMI and 86% of overweight or obese patients at the time of diagnosis were overweight or obese at the last follow-up. The difference among the groups was statistically significant (p=0.003). The comparisons of some factors for the obese, overweight, and normal-weight groups are shown in Table 3. Obesity was more frequently detected in patients who were younger than 6 years of age at diagnosis. While 6 (27%) of the survivors under 6 years of age at diagnosis were obese, none of the survivors ≥6 years of age at diagnosis were obese (p=0.023) at the last follow-up. Sex and receiving of cranial radiotherapy had no significant association with the development of overweight or obesity (p>0.005). Insulin resistance was higher in the obese group. Cumulative corticosteroid doses (as hydrocortisone equivalents) in the MRG and HRG were 14,900 mg/m2 and 22,000 mg/m2, respectively, and there was no significant difference for overweight and obesity with respect to cumulative doses between the risk groups (p=0.880). The mean height SDs of all leukemia survivors at the time of diagnosis and at the time of study were 0.89 and 0.45, respectively. The difference in the mean height SDs before and after treatment was not statistically significant (p=0.056). On the other hand, the mean height SDs were statistically lower in the group that received cranial radiotherapy (p=0.037). 

Fasting blood glucose levels of all subjects were in normal range (mean: 84.5±7 mg/dL, range: 70-98). Insulin resistance was observed in 8 (21%) subjects. Insulin resistance was more frequently seen in obese subjects and in those who had family history of type 2 diabetes. While 7 (47%) of the patients with family history of type 2 DM had insulin resistance, only 1 (4%) patient with no family history of diabetes had insulin resistance (p=0.002). Sex, age at diagnosis, treatment in the HRG, and receiving of cranial radiotherapy had no significant associations with insulin resistance (p>0.005). Hyperlipidemia was detected in 8 (21%) of the 38 survivors. Of these 8 survivors with hyperlipidemia, 4 of them had normal BMIs while the others were obese/overweight. Triglyceride and total cholesterol levels were not significantly different between subjects who were treated with cranial radiotherapy and those who received chemotherapy only. There was also no significant difference in total cholesterol and triglyceride levels between obese/overweight subjects and subjects with a normal BMI. The mean age at menarche among female survivors was 11.9±0.78 years (range: 10-13). Premature thelarche was detected in one female patient. None of the patients had short stature. Hypothyroidism was observed in one of the survivors with normal thyroid ultrasonography and negative thyroid autoantibodies. The patient with hypothyroidism was not in the HRG and did not receive cranial radiotherapy, and hypothyroidism was detected during laboratory examination without any symptoms. Two survivors had osteopenia. They were males and older than 6 years of age at the time of diagnosis. One of them was in the HRG and received 12-Gy cranial radiotherapy.

**Cardiological Late Effects**

The cardiovascular examinations, including physical examination, ECG, and echocardiogram, were normal for all patients at the time of diagnosis of ALL. Cardiovascular abnormality at follow-up was detected in only one female survivor with hypertension and cardiac diastolic dysfunction. She was 5 years old at the time of diagnosis and 13 years at the time of the study. Her blood pressure was measured as 135/85 mmHg, her E/A ratio was <1.00, and her ejection fraction was 62% at the time of the study. She was in the SRG/MRG and her cumulative anthracycline dose was 240 mg/m2. She also had obesity, hyperlipidemia, and insulin resistance.

The distribution of late effects did not differ between the SRG/MRG and the HRG (p=0.766). There was also no statistically significant difference for the distribution of late effects between subjects who did or did not receive cranial radiotherapy (p>0.005).

## DISCUSSION

Among our childhood leukemia survivors, 60% had at least 1 adverse event and 21% had multiple problems. Oeffinger et al. [3] found that 62.3% of survivors had at least 1 chronic condition. Geenen et al. [4] reported that almost 75% of survivors had 1 or more adverse events. Therapeutic exposures, including cranial radiotherapy and certain chemotherapy agents, will place ALL survivors at risk of development of serious late effects from their therapy [21]. These data emphasize the need for regular screening programs for childhood ALL survivors. The most common late effect detected in our ALL survivors was overweight/obesity. We found a high prevalence of overweight/obesity in our study, consistent with the other studies in the literature [22,23,24]. The prevalence of obese and overweight children among the general pediatric population was reported as 3.7%-6.1% and 10.3%-12.2%, respectively, in 2 different studies from Turkey [25,26]. According to these results, the prevalence of overweight/obesity detected in our population of leukemia survivors is higher than the prevalence detected in the general Turkish population.We observed that children younger than 6 years of age at diagnosis were more likely to be obese at last follow-up. In previous studies it was reported that younger children at diagnosis had a greater likelihood of becoming overweight or obese as adults than did older children at diagnosis [22,24,27]. One important risk factor for obesity in ALL is the rapid growth in adiposity and early ‘adiposity rebound’, which appears to be a typical consequence of the treatment and may explain why younger patients with ALL, particularly those with onset of the disease during the toddler and preschool years, are at the highest risk of obesity [28]. Regarding the risk factors for obesity, no significant difference was found in BMIs between patients having received cranial irradiation and those who received chemotherapy only. These findings were consistent with the findings of other studies in the literature [29,30]. There was also no significant association between high cumulative steroid dose and overweight/obesity. Further investigations with large numbers of participants are required to understand the impact of genetic background, environmental factors, and treatment components on the development of obesity in childhood cancer survivors.

Short stature and a decrease in growth velocity is a well-known side effect of acute leukemia treatment in children, especially in those who receive cranial radiotherapy. Growth-hormone deficiency is the most common endocrinopathy detected after cranial radiotherapy [21]. Consistent with these findings, in this study the mean height SD at the last follow-up was significantly lower than the mean height SD at the time of diagnosis in those who received cranial radiotherapy. On the other hand, the mean height SD of all leukemia survivors, including those who did not receive cranial radiotherapy, was also lower at the last follow-up, but the difference was not statistically significant. Although chemotherapy, and especially corticosteroids, can result in growth delay during treatment, it is known that this is normally followed by a period of catch-up growth after completion of therapy [21]. 

Insulin resistance was highly prevalent among our ALL survivors, which is in accordance with earlier studies [31,32]. Oeffinger et al. [33] reported increased prevalence of insulin resistance in young adult survivors of childhood ALL for both sexes treated with and without cranial radiotherapy. In this study, 21% of the subjects were found to have insulin resistance. Sex, treatment with cranial radiotherapy, and BMI were not significantly associated with insulin resistance. We observed that obese subjects and those with family history of type 2 diabetes were more likely to have insulin resistance. The development of insulin resistance is multifactorial. Increased rates of insulin resistance in leukemia survivors may be the result of interactions between familial background and environmental factors such as treatment components or nutritional status.

Hyperlipidemia was detected in 8 (21%) of the 38 survivors. Survivors of childhood ALL are at risk for visceral obesity, dyslipidemia, insulin resistance, and hypertension [32,34,35]. These features of metabolic syndrome are linked to growth-hormone deficiency and in turn to cranial radiotherapy [36,37]. However, obesity and metabolic syndrome components (as well as growth-hormone deficiency) have also been described in patients treated with chemotherapy only [38]. 

In our study, none of the survivors were found to have short stature. Growth-hormone deficiency is reported as the most common endocrinopathy detected after cranial radiotherapy [39]. It is more frequent with radiotherapy doses higher than 24 Gy, but it has been observed with doses as low as 18 or 10 Gy given as a single dose as part of total-body irradiation [39,40]. In addition to higher cranial radiotherapy doses, risk factors for growth-hormone deficiency and short stature include younger age at diagnosis and female sex [39,40,41]. The reason for not detecting short stature in our patients might be due to lower cranial radiotherapy doses (≤18 Gy). 

Precocious puberty is a well-described late effect of cranial radiotherapy at doses of 18 to 24 Gy, and is more common in girls [42,43,44]. However, most female ALL survivors experience menarche at a normal age [45,46]. Premature thelarche was detected in one survivor in our study. The small number of events in this study might be due to relatively lower cranial radiotherapy doses. The mean age at menarche among our ALL survivors was 11.9±0.78 years. Other studies from Turkey reported the mean age at menarche as 12.4 and 13.04, respectively [47,48]. The mean self-reported age of our leukemia survivors at menarche was similar to Turkish population norms. 

Studies that have examined thyroid dysfunction following ALL therapy have either reported no significant thyroid function abnormalities [49,50] or more subtle compensated hypothyroidism [51,52,53]. The Childhood Cancer Survivor Study Group reported that cranial radiotherapy alone was insufficient to induce reportable hypothyroidism, whether central or primary in origin. On the other hand, they also noted that craniospinal radiotherapy used in ALL was associated with a higher amount of thyroid gland exposure and was strongly associated with subsequent hypothyroidism [54]. In our study, none of the patients had received craniospinal radiotherapy, and hypothyroidism was detected in only one patient of the MRG who had not received cranial radiotherapy. 

Among our ALL survivors, 2 were found to have osteopenia. Osteonecrosis (avascular necrosis) and decreased BMD are both reported to be associated with treatment of ALL, specifically with prolonged corticosteroid exposure [21]. Decreased BMD is known to be caused by a number of factors, including the disease itself, concurrent serious infections, poor nutrition, decreased physical activity, and abnormalities in vitamin D metabolism, in addition to various components of treatment (especially glucocorticoids) and radiotherapy [55]. Halton et al. [56] found that BMD diminished during the first 2 years of chemotherapy in patients older than 11 years old at the time of diagnosis but remained stable in those who were younger. Both Van der Sluis et al. [57] and Lequin et al. [58] also reported no significant long-term effects on height, BMD, or lean body mass in children with ALL treated with high-dose dexamethasone and methotrexate but not with cranial irradiation. The small number of cases of osteopenia in this study might be related to the younger age of the subjects at diagnosis (84% of them were <10 years of age at diagnosis) and the relatively lower doses of cranial radiotherapy. Only 3 (8%) of the subjects received 18 Gy of cranial radiotherapy and 13 (34%) of them received 12 Gy.

In this study, clinical cardiovascular abnormality was detected in one subject with hypertension and cardiac diastolic dysfunction. Through a variety of different mechanisms, it appears that survivors of childhood ALL have an increased prevalence of several cardiovascular risk factors and thus are at increased risk for developing cardiovascular disease. Cardiotoxicity is associated with exposure to anthracyclines and irradiation to the heart, particularly at a young age [59,60]. Doses of ≥300 mg/m2 of doxorubicin or daunorubicin are associated with the highest risk of cardiotoxicity, usually in the form of left ventricular dysfunction [61]. ALL survivors, particularly those who receive more than 24 Gy of cranial radiotherapy, may be at an even further increased risk for cardiac events due to additional cardiovascular risk factors like obesity, physical inactivity, and metabolic syndrome [62]. In a study from Turkey including 55 ALL survivors and 38 healthy controls with a mean follow-up time of 34.1±21 months, no overt cardiotoxicity was reported [63]. Rammeloo et al. [64] compared cardiac functions in ALL survivors who were treated with 100 mg/m2 of daunorubicin and in survivors who were not treated with anthracycline. They detected no difference in left ventricular functions between the 2 groups. In our study, HRG patients (34%) received 300 mg/m2 anthracycline and the remaining (66%) had lower doses. The small number of cardiovascular events in this study might be due to the moderate doses of anthracyclines and a relatively shorter follow-up time with an average of 8 years, since it is well established that the heart compensates for about 5-15 years following therapy in most survivors with anthracycline-induced cardiomyopathy [60]. 

 There are some limitations of this study. One limitation is the relatively small size of the study group. Further investigations with large numbers of participants are needed for detection of the late effects in a wide spectrum in leukemia survivors. Another limitation is related to the adequacy of the methods used to evaluate cardiological late effects. The methods that we performed to evaluate cardiological late effects in this study were not sensitive enough to detect subclinical cases. There are some other methods reported in the literature to evaluate subclinical cardiological toxicity, including tissue Doppler imaging, myocardial performance index, and some biomarkers like B-type natriuretic peptide (BNP) and proBNP [63,65,66]. A more comprehensive, detailed detection of cardiological late effects including subclinical changes could have been performed if we had been able to use these mentioned methods. 

In conclusion, 60% of ALL survivors had at least one endocrinological or cardiological late effect. Survivors of childhood leukemia are at risk of developing endocrinological and cardiological late effects as a result of exposure to chemotherapy and radiotherapy. Therefore, long-term, regular, and comprehensive follow-up of childhood ALL survivors is recommended for early recognition of these late effects and to prevent development of worse outcomes. 

## CONFLICT OF INTEREST STATEMENT

The authors have no conflicts of interest to declare. 

## Figures and Tables

**Table 1:**
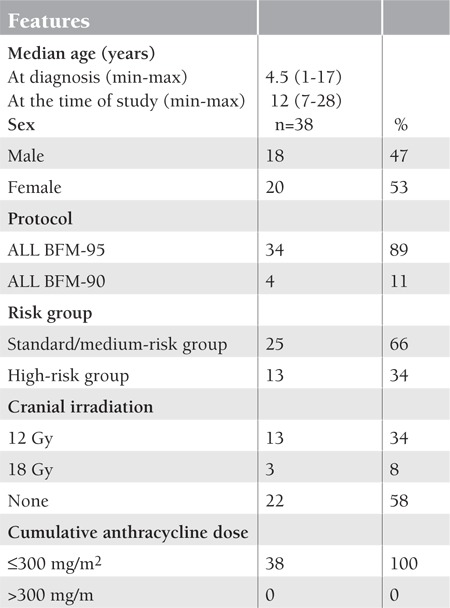
The characteristics of 38 leukemia survivors

**Table 2:**
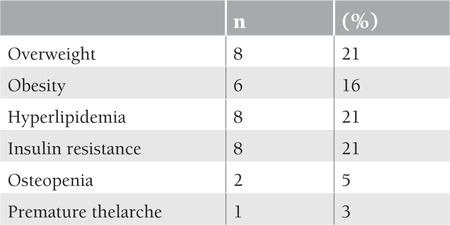
Late effects in acute lymphoblastic leukemia survivors.

**Table 3:**
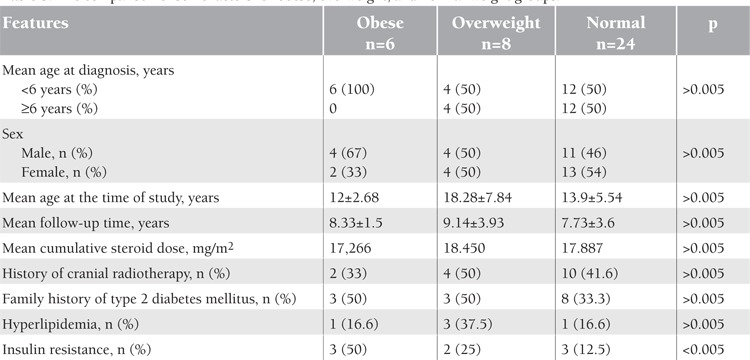
The comparison of some factors for obese, overweight, and normal-weight groups
